# Meta-Analysis—Correlation between Spiral Ganglion Cell Counts and Speech Perception with a Cochlear Implant

**DOI:** 10.3390/audiolres11020020

**Published:** 2021-05-26

**Authors:** Yew-Song Cheng, Mario A. Svirsky

**Affiliations:** 1 Department of Otolaryngology, New York University School of Medicine, New York, NY 10016, USA; yewsong.cheng@nyulangone.org; 2 Skirball Institute for Biomolecular Medicine, New York University School of Medicine, New York, NY 10016, USA; 3 Neuroscience Institute, New York University School of Medicine, New York, NY 10016, USA; 4 Department of Neuroscience and Physiology, New York University School of Medicine, New York, NY 10016, USA

**Keywords:** cochlear implants, speech perception, spiral ganglion cells

## Abstract

The presence of spiral ganglion cells (SGCs) is widely accepted to be a prerequisite for successful speech perception with a cochlear implant (CI), because SGCs provide the only known conduit between the implant electrode and the central auditory system. By extension, it has been hypothesized that the number of SGCs might be an important factor in CI outcomes. An impressive body of work has been published on findings from the laborious process of collecting temporal bones from CI users and counting the number of SGCs to correlate those numbers with speech perception scores, but the findings thus far have been conflicting. We performed a meta-analysis of all published studies with the hope that combining existing data may help us reach a more definitive conclusion about the relationship between SGC count and speech perception scores in adults.

## 1. Introduction

Cochlear implants represent the most successful example of a neural prosthesis that replaces a human sense with an electronic device. They do so by stimulating the auditory nerve directly, bypassing the normal mechanism that transduces sound into neural activity [1]. However, clinical outcomes can be variable, with some patients achieving much higher levels of speech perception than others, which has driven continued interest in identifying factors that might be responsible for this variability. For example, demographic variables such as longer duration of profound bilateral deafness [2,3], older age at implantation [4], and lower levels of either preoperative [5,6] or postoperative residual hearing in the implanted ear [7] have been associated with poorer outcomes.

Physiological and anatomical variables have also been suggested as possible factors underlying individual differences [8]. In particular, the number of remaining spiral ganglion cells (SGCs) is frequently cited as a potentially important factor [9,10]. This is a very reasonable hypothesis because SGCs (including the soma and the peripheral axons) are almost certainly the sole conduit by which electrical pulses generated by the cochlear implant can elicit activity in the auditory system. If a patient has complete absence of SGCs, a cochlear implant is likely unable to elicit the action potentials required to propagate the signal along the cochlear nerve to deliver information to the auditory system. Therefore, it is quite plausible that a healthier auditory nerve (indexed by a larger number of remaining SGCs) might be associated with better outcomes for cochlear implant patients.

Obtaining experimental support for this hypothesis is complicated because there is no reliable, precise, and noninvasive way to count the number of SGCs in live human subjects. The most direct way to establish the correlation between SGCs and CI outcomes is therefore to measure those outcomes (particularly speech perception scores, which are deemed the most important outcomes of cochlear implantation in adults) while patients are alive, and then analyze their temporal bones post-mortem to obtain precise SGC counts. Dedicated groups of otohistopathologists have taken on this challenging question and have published data on SGC counts and speech perception scores in CI users. However, results have been conflicting, and the first two studies to compare SGC counts and speech performance in patients who had used multichannel CIs found an unexpected negative correlation between CI speech perception scores and SGC counts [11,12], which is contrary to the prevailing hypothesis.

Here, we performed a meta-analysis of all published studies of the relationship between SGC count and speech perception scores in adult patients who received a multichannel cochlear implant, to help us reach more solid conclusions about an unconfirmed but widely accepted hypothesis within the cochlear implant field.

## 2. Materials and Methods

We followed the Preferred Reporting Items for Systematic Reviews and Meta-analyses (PRISMA) checklist to guide our study [13]. This study was not pre-registered.

The population of interest was post-lingually deaf subjects who received the intervention of cochlear implantation. Most subjects had either consonant-nucleus-consonant (CNC) or Northwestern University (NU-6) word scores, two monosyllabic word identification tests that have been shown to result in “remarkably similar” scores [14]. In 5 subjects where CNC or NU-6 was not available, Nadol et al., Khan et al., Li et al. and Kamakura et al. [12,15,16,17] reported estimated NU-6 or CNC scores. This was done based on the work of Rabinowitz et al. [18], who found that NU-6 word identification scores are highly predictable based on CUNY (City University of New York) sentence identification scores (the Pearson correlation between predicted and measured scores was +0.93). Khan et al., Li et al. and Kamakura et al. based their predictions on scores from the HINT (Hearing in Noise Test) or CID (Central Institute for the Deaf) tests rather than the CUNY sentence test, but these sentence tests are similar enough to make the predictions quite reasonable. In fact, a sensitivity analysis showed that NU-6 scores could be predicted within a 15 percentage point window based on HINT or CID scores. Thus, following the lead of these authors, we applied the same estimation from CID sentence scores to four subjects from Xu et al., 2012 and Ishiyama et al., 2019 [19,20]. Subjects from Ishiyama et al., 2019 who only had speech perception data from tests other than CNC, NU-6, or CID sentences were excluded from the analysis.

**Figure 1:**
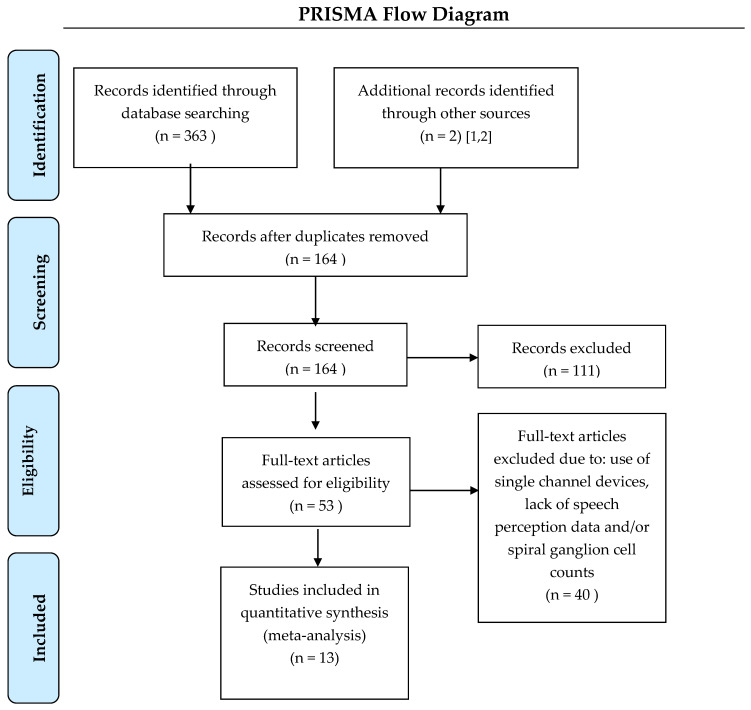
PRISMA 2009 flow diagram. A total of 13 studies were included in this quantitative synthesis [11,12,15,16,17,19,20,21,22,23,24,25,26].

The literature search was performed by a medical librarian for studies reporting spiral ganglion cell counts and speech perception scores in patients with cochlear implants. The search strategy used keywords and controlled vocabulary terms (for example, “cochlear implants”, “spiral ganglion”, and “temporal bone”; the full search strategy is listed as supplementary information) and was executed starting at the earliest journal articles in each database up to January of 2021 on PubMed/Medline, Biosis, Embase, and Web of Science. A PRISMA flow diagram (Figure 1) details the search outcomes.

The research abstracts were reviewed independently by both authors (YSC and MAS). Studies reporting (1) speech perception scores and (2) total spiral ganglion cell counts in human subjects using multichannel cochlear implants were included in this study. In addition to word recognition scores and total SGC counts, available patient demographic information, type of implant, depth of insertion and presumed etiology of deafness was collected.

### Statistical Analyses

Linear regressions of speech perception scores as a function of number of spiral ganglion cells were performed using Prism 9 (Graphpad, San Diego, CA, USA) for the whole dataset. Then, the process was repeated for two subsets of the data: studies conducted at Massachusetts Eye and Ear Infirmary (MEEI) and studies conducted at other institutes. Significance was evaluated before and after Bonferroni corrections to account for the fact that three tests were run, i.e., the original data set and the two subsets. Lastly, Fisher’s exact test was used to compare the proportion of patients with speech scores above 50% correct among those below or above a certain threshold of spiral ganglion cell count. This was done for two threshold values: 2800 and 3000 spiral ganglion cells.

In addition to the univariate analyses listed above, several multivariate analyses were conducted to see if a relation between speech perception scores and number of spiral ganglion cells was present when including other independent variables in the analysis. In total, six multivariate linear regressions were conducted with the corresponding independent variables being: number of spiral ganglion cells (#SGC) and age at implantation; #SGC and age at death; #SGC and duration of deafness; #SGC and duration of implantation; #SGC, age at implantation, and age at death; #SGC and age at implantation, age at death, and duration of deafness.

## 3. Results

A total of 13 studies were found to contain relevant data [11,12,15,16,17,19,20,21,22,23,24,25,26], of which 10 studies were from MEEI [12,15,16,17,21,22,23,24,25,26]. Duplicate subjects were identified and removed from the analysis, which left a total of 81 subjects. We also excluded nine subjects who had electrode insertions under 15 mm (which are known to result in poorer speech perception outcomes regardless of other factors) and 12 subjects with unknown insertion depths. Thus, 60 subjects were included in the final analysis. Results were unaffected by including or excluding subjects with unknown insertion depths. Eighteen subjects had their temporal bones evaluated at two separate time points and the SGC counts were different, as might be expected. When differences were less than 20% (which happened in 12 cases) we selected the most recent count. In cases where SGC counts were markedly different, we performed our analysis with both sets of data and found that discrepancies did not alter the conclusions of our findings. Having established this, it was decided that the most recent total SGC counts would be used in this study.

**Figure 2:**
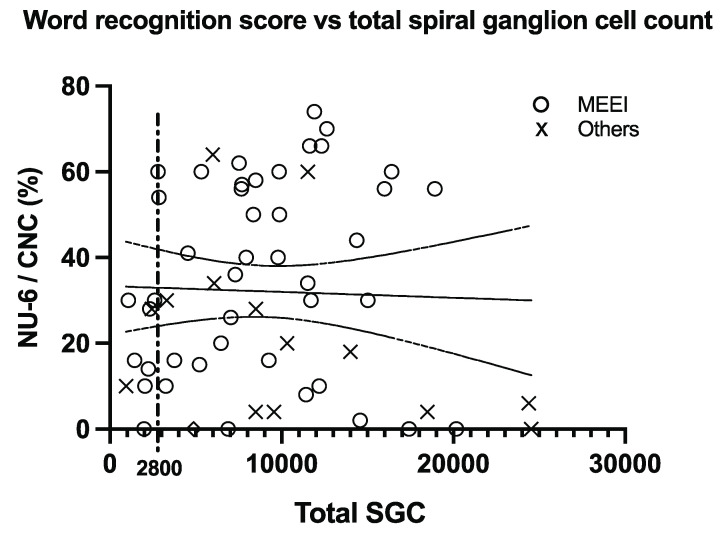
Speech perception scores as a function of total number of spiral ganglion cells in patients examined at MEEI (circles) or other sites (X). The vertical line at SGC count = 2800 helps visualize that subjects below that level tend to have lower speech perception scores.

Figure 2 shows a scatterplot of the whole data set. There was no significant correlation between speech scores and the number of spiral ganglion cells (the p-value for the regression slope was 0.80). The MEEI data subset did show a modest correlation when including data from subjects with shallow electrode insertions, or insertions of unknown length (r = +0.290, adjusted r^2^ = 0.084, *p* = 0.030), but this became insignificant after Bonferroni correction or after removing the data points with shallow or unknown insertion lengths.

Post-hoc examination of the whole data set showed that 32% (19 out of 60) of the subjects who had SGC counts above 2800 scored above 50% correct in the word identification test, while none of the nine subjects with SGC counts below 2800 did. Fisher’s exact test comparing these two proportions (32% and 0%) yielded a significant result (*p* = 0.0463). However, the significant result disappeared (*p* = 0.476) when a threshold of 3000 rather than 2800 SGCs was used.

Lastly, none of the six multivariate linear regressions modified the findings observed in the univariate analysis. The only regression parameter whose point estimate was significantly different from zero, for any of these regressions, was the intercept. In particular, no significant relation between SGC count and speech perception scores was observed.

## 4. Discussion

The most important result of the present analysis was a negative one: no significant correlation between SGC count and speech perception scores was found shows the absence of a correlation. The planned analysis (linear regression for the whole data set) yielded no compelling evidence of a systematic relationship between SGC count and speech perception scores. Some post-hoc analyses did result in statistically significant findings, but these disappeared after correcting for multiple comparisons or making slight changes in analysis parameters. An interpretation of our results is that the hypothesized relationship between SGC count does not exist. However, this would be a surprising result because spiral ganglion cells are the only known neurons that are stimulated by set word identification scores to approach zero.

One possible explanation for the absence of correlation in our analysis is that a small number of SGCs may be sufficient to deliver the information conveyed via electrical stimulation by a cochlear implant, and speech perception is only affected at very low SGC counts. This would be consistent with the observation that the nine patients with an SGC count below 2800 failed to score above 50% words correct. However, this observation is completely exploratory, based on a post-hoc analysis, and it is highly dependent on the specific SGC count threshold that is chosen for the analysis. This result illustrates the point that one should be wary of post-hoc analyses that were conducted after having seen the data, instead of being determined in advance and pre-registered. Nonetheless, and consistent with this explanation, Otte, Schuknecht and Kerr looked at 41 human temporal bones and established that speech discrimination (with acoustic hearing) required at least 10,000 SGCs [27]; Blamey, in 1997, hypothesized that the minimum number of SGCs required for CIs to work may be quite low and that most CI recipients exceed this threshold [28], a hypothesis that the present analysis supports.

Other possible explanations for the lack of correlation between SGCs and speech perception scores have been discussed in the studies included in our meta-analysis. These include varied etiology of hearing loss within the study population, and widely ranging intervals between time of last audiology test and death. Cognitive function at the time of testing is also not known, and it could have an impact on speech perception scores. Other known factors such as duration of bilateral deafness and compliance with using the CI every day are not taken into account either.

To control for confounding factors, Seyyedi et al. examined within-subject correlations in bilateral CI users by comparing inter-aural differences in SGC counts to inter-aural differences in word identification scores for six patients [23]. A weak but statistically significant positive correlation was found. However, that correlation depended on a single, highly atypical implanted ear from a single patient (Patient 1). The left ear in that patient outperformed the right ear (25% vs. 5%) and did have a greater SGC count, but there were other important left–right differences: the left ear had been implanted for 11 years versus only 1 year for the right, and the depth of insertion was almost twice for the left ear than for the right (11 mm vs. 6 mm). This is important because speech perception with multichannel CIs is influenced by extreme values of insertion depth. Average word identification scores with the CI alone, with standard insertion depth values (at least 20–25 mm) are above 60% [29], Hybrid S8 patients (whose electrodes are 10 mm long) score 30% on average and, lastly, the rare multichannel CI users whose insertion depths do not exceed 6 mm typically show zero scores [30] or are reported to have very poor speech perception performance (CNC scores in the CI-alone condition unreported) [31]. In our own analysis of the data from Seyyedi et al., when Patient 1 was removed, the reported correlation was no longer significant. Similarly, the positive correlation seen in this meta-analysis within the pooled MEEI data failed to be statistically significant when shallow insertions were excluded.

## 5. Conclusions

After examining all available published data, the present meta-analysis did not find a significant correlation between SGC counts and word recognition scores in CI users. This result is consistent with the possibility that a relatively small number of SGCs is sufficient to understand speech with a cochlear implant, and therefore the correlation in question does not exist for patients with SGC counts between 1000 and 25000 (the range found in this study). The absence of a correlation is also consistent with the possibility that any influence of SGC count on speech perception by CI users is obscured by other factors that are more strongly associated with speech perception outcomes.

## Data Availability

Dataset used to general reported results was submitted as supplementary material.

## Supplementary Materials

The following are available online at https://www.mdpi.com/article/10.3390/audiolres11020020/s1.
